# Prognostic significance of patent foramen ovale in anticoagulated patients with atrial fibrillation

**DOI:** 10.1136/openhrt-2019-001229

**Published:** 2020-04-06

**Authors:** Rowlens M Melduni, Waldemar E Wysokinski, Zhenzhen Wang, Bernard J Gersh, Samuel J Asirvatham, Sri Harsha Patlolla, Eddie L Greene, Jae K Oh, Hon-Chi Lee

**Affiliations:** 1 Cardiovascular Medicine, Mayo Clinic, Rochester, Minnesota, USA; 2 Medicine, Division of Nephrology and Hypertension, Mayo Clinic College of Medicine, Rochester, Minnesota, USA

**Keywords:** electrocardiography, atrial fibrillation, atrial flutter, stroke, echocardiography

## Abstract

**Objective:**

Previous studies have postulated a causal role of patent foramen ovale (PFO) in the aetiology of embolic stroke in the general population. We hypothesised that the presence of concomitant PFO and atrial fibrillation (AF) will add incremental risk of ischaemic stroke to that linked to AF alone.

**Methods:**

We analysed data on 3069 consecutive patients (mean age 69.4±12.2 years; 67.1% men) undergoing transoesophageal echocardiography-guided electrical cardioversion (ECV) for AF between May 2000 and March 2012. PFO was identified by colour Doppler and agitated saline contrast study. All patients were followed up after ECV for first documentation of ischaemic stroke. Outcomes were compared using Cox regression models.

**Results:**

The prevalence of PFO was 20.0% and the shunt direction was left-to-right in the majority of patients (71.4%). Patients with PFO had a higher frequency of obstructive sleep apnoea (21.7% vs 17.1%, p=0.01) and higher mean peak left atrial appendage emptying velocity (38.3±21.8 vs 36.1±20.4 cm/s; p=0.04) compared with those without PFO. Otherwise, baseline characteristics were similar between groups. During a mean follow-up period of 7.3±4.6 years, 214 patients (7.0%) had ischaemic stroke. Multivariable analysis showed no significant association between PFO and ischaemic stroke (HR, 0.82 (95% CI 0.57 to 1.18)). PFO shunt direction was strongly associated with stroke: HR, 1.91 (95% CI 1.16 to 3.16) for right-to-left shunt and HR, 0.58 (95% CI 0.36 to 0.93) for left-to-right shunt.

**Conclusions:**

The presence of concurrent PFO in this largely anticoagulated group of patients with AF was not associated with increased risk of ischaemic stroke.

**Key questions**

**What is already known about this subject?**

- The literature suggests that the presence of a patent foramen ovale (PFO) may have a role in the pathogenesis of cerebral and systemic embolism ostensibly because of paradoxical embolisation from sources within the venous circulation across the atrial right-to-left shunt.
- Atrial fibrillation (AF) is also a condition that is linked to ischaemic stroke. Yet, it is not known whether the coexistence of PFO and AF has incremental impact on the risk of ischaemic stroke compared with either condition alone.

**What does this study add?**

- Although previous studies have postulated a causal role of PFO in the aetiology of embolic stroke in the general population, in the present study, the presence of PFO in patients with AF was not associated with embolic risk beyond those with right-to-left shunt and other established risk factors in this predominantly anticoagulated AF population. Large-scale studies are needed to further examine the impact of PFO and shunt flow direction on clinical outcomes in AF patients.
- The presence of concurrent PFO does not increase the risk of ischaemic stroke in patients with AF, mainly due to shunt flow at baseline (unprovoked) being directed from left-to-right in the majority of patients with AF.

**How might this impact on clinical practice?**

- This study’s finding of no association of PFO with stroke in patients with AF suggests that in patients with no competitive stroke risks, adequate oral anticoagulation with or without concurrent antiplatelet therapy may suffice to mitigate the purported PFO-stroke association.

## Introduction

Patent foramen ovale (PFO) is a common remnant of the foetal circulation that is found in about 25% to 30% of the general population.^1 2^ Previous studies have shown that the presence of a PFO may have a role in the pathogenesis of cerebral and systemic embolism, ostensibly because of paradoxical embolisation from sources within the venous circulation across the atrial right-to-left shunt.^3–5^ Several case-control and population-based studies, including a meta-analysis of these studies have reported a significant association between PFO and cryptogenic ischaemic strokes.^6–8^

Similarly, atrial fibrillation (AF) is a condition that is also linked to ischaemic stroke.^9^ Despite the recognised increased risk of cerebrovascular events associated with both PFO and AF, it is not known whether the coexistence of PFO and AF has an incremental impact on the risk of ischaemic stroke. We hypothesise that the concomitant presence of PFO and AF would pose a larger risk of stroke compared with either condition alone. Therefore, the purpose of this study was to examine whether the presence of a PFO influences the risk of ischaemic stroke in patients with AF.

## Methods

### Study design and population

We analysed prospectively collected data on all patients who underwent their first transoesophageal echocardiography (TEE)-guided electrical cardioversion for persistent AF at the Mayo Clinic between May 2000 and March 2012 (n=3329). Patients were excluded if they had a history of congenital heart disease, moderate or greater mitral stenosis, mitral valve surgery or no documented assessment of interatrial shunting (n=260). A total of 3069 patients comprised the study population.

### Indirect patient and public involvement

We did not directly include patient and public involvement (PPI) in this study, but the database used in the study was developed with PPI and is updated by a committee that includes patient representatives.

### Echocardiography imaging and electrical cardioversion protocol

All patients underwent TEE immediately before electrical cardioversion to exclude atrial thrombus, according to a previously described protocol.^10 11^ Patients were routinely assessed for PFO and direction of shunt flow at the atrial level according to guidelines of the American Society of Echocardiography.^12^ Sequential interrogation was performed using standard views from 0 to 90 degrees. The colour Doppler scale was reduced to document low-velocity flow across atrial communication if present. Agitated saline combined with provocative physiological manoeuvres was also performed to transiently increase right atrial pressure and provoke right-to-left shunting. Evaluation of left ventricular (LV) ejection fraction and left atrial (LA) enlargement were based on semi-quantitative visual assessment by TEE examination.

### Definitions

#### Patent foramen ovale

A PFO was defined as shunting of blood across the interatrial septum seen on colour flow Doppler and/or the appearance of microbubbles in the LA or LV within three cardiac cycles after opacification of the right atrium with intravenous injection of agitated saline either at rest or after cough, and/or Valsalva release.

#### Outcome ascertainment

The primary outcome was ischaemic stroke, determined on the basis of results of radiographic examination, including MRI or CT scan or by physician decision after thorough chart reviews. Stroke outcomes were verified from the health records by cross reference with available administrative databases and outside records, when possible.

### Statistical analysis

Continuous variables are presented as means±SD and median (IQR), as appropriate and categorical variables as counts and percentages. Comparisons between groups with and without PFO were performed using the t-test for continuous variables and χ2 test for categorical variables. Kaplan-Meier survival analyses with log-rank tests were used to estimate cumulative probabilities of ischaemic stroke on the basis of all available follow-up data among PFO and non-PFO groups. Patients who did not experience the event of interest (ischaemic stroke) during follow-up were censored on the follow-up end date or death. A time-dependent Cox proportional hazard regression model was used to estimate the independent association of PFO and other predictors with ischaemic stroke based on clinical variables at the time of TEE-guided cardioversion. Assessment of prognostic variables was first performed by univariate analysis and baseline covariates with a p value ≤0.10 were included in the multivariable model. The potential confounding variables included in the multivariable regression model were CHA_2_DS_2_-VASc score (congestive heart failure, hypertension, age ≥75 (doubled), diabetes mellitus, prior stroke or transient ischaemic attack (doubled), vascular disease, age 65 to 74, female), mean left atrial appendage (LAA) emptying velocity, LAA thrombus, mitral regurgitation (≥moderate) and patent foramen ovale. For assessment of the impact of PFO shunt directionality on the risk of stroke, in lieu of patent foramen ovale, the variables ‘PFO with right-to-left shunt’ and ‘PFO with left-to-right shunt’ were tested separately versus no PFO. For each variable, HR and 95% CI were computed. For proportional hazards models, assumptions were tested by analysing main effects and product terms of covariates and time factor transformed to logarithmic scale. Data for ties were approximated using the Efron method. All tests of significance were two-tailed, and an α level of 0.05 was considered the threshold for statistical significance. All statistical analyses were performed using SAS software V.9.4 (SAS Institute Inc, Cary, North Carolina).

## Results

### Population characteristics

Baseline characteristics of the patient population are presented in table 1. The overall age of the patient population ranged from 18 to 98 years (mean, 69.4±12.2; median (IQR) 71 (62 to 78) years), 13.6% were younger than 55 years of age, 27.0% were younger than 65 years and 67.1% were men. The prevalence of PFO among the 3069 patients studied was 20.0%. The PFO shunt flow direction at baseline (ie, unprovoked) was left-to-right in 71.4%, right-to-left in 24.3% and bidirectional in 4.3% of patients and 46.9% had provoked right-to-left shunt. Patients with PFO had a higher frequency of obstructive sleep apnoea (OSA) (21.7% vs 17.2%, p=0.01), a higher mean peak left atrial appendage emptying velocity (LAAEV) (38.3±21.8 vs 36.1±20.4 cm/s, p=0.04) and a non-significant tendency toward higher body mass index (31.3±6.9 vs 30.7±6.7 kg/m^2^, p=0.09). Otherwise, baseline characteristics including age, sex, prior history of stroke (12.1% vs 11.2%, p=0.57), CHA_2_DS_2_-VASc score, left ventricular ejection fraction, duration of AF, oral anticoagulation (OAC) therapy and antiplatelet therapy were similar between the two groups.

**Table 1:** Baseline characteristics*

Characteristic	No PFO	PFO	P value
	n=2455 (80.0%)	n=614 (20.0%)	
**Demographics**			
Age, years	69.4±12.2	69.3±12.0	0.92
Age >75 years	936 (38.1)	219 (35.7)	0.26
Sex (male)	1655 (67.4)	405 (66.0)	0.49
Body mass index, kg/m^2^	30.7±6.8	31.3±6.9	0.09
**History variables**			
Hypertension	1682 (68.8)	409 (66.6)	0.30
Diabetes mellitus	501 (20.5)	126 (20.6)	0.95
Prior myocardial infarction	403 (16.6)	94 (15.6)	0.57
Prior CABG	354 (14.5)	81 (13.3)	0.45
Stroke or TIA	276 (11.2)	74 (12.1)	0.57
Congestive heart failure	1041 (42.4)	263 (42.8)	0.85
Cardiomyopathy (tachycardia induced, ischaemic, dilated, infiltrative, restrictive, hypertrophic or idiopathic)	584 (23.8)	146 (23.8)	0.99
Smoking (current or former)	1169 (47.6)	308 (50.2)	0.26
Chronic lung disease	334 (13.7)	84 (13.8)	0.94
Obstructive sleep apnoea	420 (17.1)	133 (21.7)	0.01
CHA_2_DS_2_-VASc score	3.2±1.6	3.2±1.6	0.60
Peripheral arterial disease	273 (11.1)	70 (11.4)	0.84
**Preprocedural haemodynamics**			
Heart rate	92.9±22.8	92.6±22.0	0.81
Duration of AF episode			
<48 hour	323 (15.4)	63 (12.2)	0.23
>2 days to <7 days	585 (27.9)	155 (30.1)	
>7 days to <1 year	1119 (53.41)	267 (51.8)	
>1 year	68 (3.25)	30 (5.83)	
**Preprocedural medications**			
Beta blocker	1511 (61.7)	354 (58.1)	0.11
Calcium channel blocker (non-dihydropyridine)	846 (34.5)	188 (30.8)	0.08
Statin	830 (33.8)	190 (30.9)	0.18
ACE-I or ARB	1039 (42.3)	255 (41.5)	0.72
Antiplatelets	1349 (55.0)	337 (54.9)	0.98
Warfarin or NOACs	756 (30.8)	202 (32.9)	0.32
Antiarrhythmics	768 (31.3)	199 (32.4)	0.59
**Echocardiography**			
*Patent foramen ovale shunt direction*			<0.001
*No PFO	–	–	–
Right-to-left		148 (24.34)	
Left-to-right		434 (71.38)	
Bidirectional		26 (4.28)	
Mean LAA emptying velocity	36.1±20.4	38.3±21.1	0.04
Spontaneous echo contrast (LA or LAA)	1082 (51.3)	277 (54.4)	0.21
Left atrial appendage thrombus	34 (1.4)	10 (1.6)	0.65
Severe left atrial enlargement	907 (37.0)	210 (34.2)	0.20
LV ejection fraction	51.2±14.0	51.9±13.4	0.28
Mitral regurgitation (≥moderate)	631 (25.7)	164 (26.7)	0.61
**Discharge medications**			
Beta blocker	1485 (60.8)	349 (57.3)	0.11
Calcium channel blocker (non-dihydropyridine)	457 (18.8)	122 (20.0)	0.48
Statin	903 (36.8)	203 (33.1)	0.08
ACE-I or ARB	1200 (48.9)	272 (44.3)	0.04
Antiplatelets	1286 (52.4)	321 (52.3)	0.96
Warfarin or NOACs	2161 (88.0)	552 (89.9)	0.19
Antiarrhythmics	829 (33.8)	223 (36.3)	0.24
Preprocedural INR	1.9±0.9	1.9±0.8	0.39
INR at discharge	1.9±0.8	2.0±0.8	0.72

* denotes reference category

ACE-I, angiotensin-converting enzyme inhibitor; AF, atrial fibrillation; ARB, angiotensin-receptor blocker; CABG, coronary artery bypass grafting; CHA_2_DS_2_-VASc, congestive heart failure, hypertension, age ≥75 (doubled), diabetes mellitus, prior stroke or transient ischaemic attack (doubled), vascular disease, age 65 to 74, female; INR, international normalised ratio; LA, left atrial; LAA, left atrial appendage; LV, left ventricular; NOACs, novel oral anticoagulants; PFO, patent foramen ovale; TIA, transient ischaemic attack.

Cardioversion success rate was not different between patients with PFO and those without (99.3% vs 99.2%, p=0.76).

### Outcomes

#### PFO and ischaemic stroke

**Figure 1:**
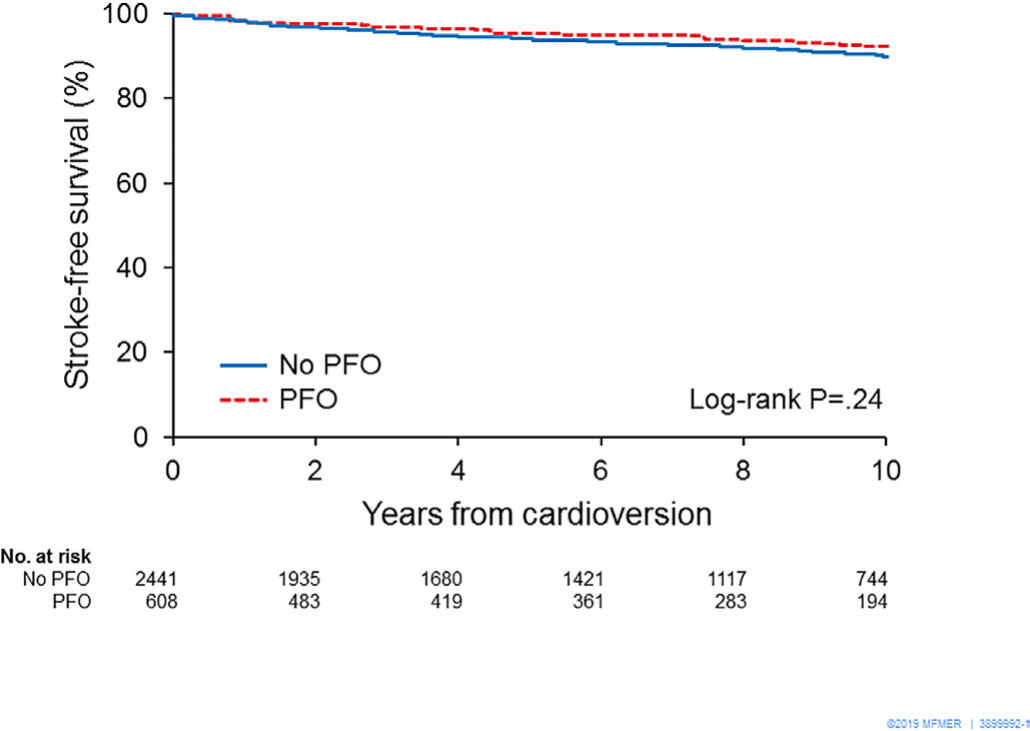
Kaplan-Meier survival analysis showing freedom from ischaemic stroke in patients with versus without PFO. The survival curves show no significant difference in the probability of stroke-free survival between patients with and without PFO. PFO, patent foramen ovale.

Follow-up was complete for all patients. The patients were observed for a mean of 7.3±4.6 years. During the follow-up period, 214 (7.0%) patients had ischaemic strokes. In the approximately 10% of patients who were not discharged on oral anticoagulation, there were 3/28 strokes in the PFO group versus 21/186 strokes in the no-PFO group, p=0.10. Kaplan-Meier survival analysis showed no significant difference in the probability of stroke-free survival between patients with and without PFO (log-rank p=0.24) (figure 1). Multivariable Cox regression analysis showed no significant association between PFO and ischaemic stroke in both crude (HR, 0.81 (95% CI 0.57 to 1.16)) (table 2) and adjusted analysis (HR, 0.82 (95% CI 0.57 to 1.18)), respectively. Independent predictors of stroke were CHA_2_DS_2_-VASc score (HR, 1.31, (95% CI 1.19 to 1.43)), and LAA thrombus (HR, 2.07, (95% CI 1.11 to 4.45)) (table 3A).

**Table 2:** Univariate Cox regression analysis to identify predictors of ischaemic stroke

Characteristic	Ischaemic stroke	
	HR (95% CI)	P value
**Demographics**		
Age (years)	1.04 (1.03 to 1.05)	<0.001
Age >75 years	1.89 (1.44 to 2.48)	<0.001
Sex (male)	0.74 (0.56 to 0.97)	0.03
Body mass index	0.99 (0.97 to 1.01)	0.37
**History variables**		
Hypertension	1.46 (1.08 to 1.98)	0.02
Diabetes mellitus	1.13 (0.81 to 1.58)	0.48
Prior myocardial infarction	1.46 (1.04 to 2.07)	0.03
Prior CABG	1.56 (1.09 to 2.22)	0.01
Stroke or TIA	2.54 (1.82 to 3.53)	<0.001
Congestive heart failure	1.19 (0.90 to 1.56)	0.22
Cardiomyopathy	0.64 (0.45 to 0.92)	0.02
Smoking (current or former)	1.00 (0.76 to 1.31)	0.99
Chronic lung disease	0.99 (0.64 to 1.52)	0.95
Obstructive sleep apnoea	1.02 (0.72 to 1.45)	0.91
Peripheral arterial disease	1.46 (0.98 to 2.17)	0.06
CHA_2_DS_2_-VASc score	1.32 (1.21 to 1.44)	<0.001
**Preprocedural haemodynamics**		
Heart rate	1.00 (0.99 to 1.01)	0.67
Duration of AF episode	–	0.32
<48 hour	0.97 (0.71 to 1.31)	0.83
<24 hours	2.72 (0.83 to 8.89)	0.10
24–48 hours	2.37 (0.74 to 7.57)	0.15
>2 days to <7 days	2.15 (0.68 to 6.77)	0.19
**Preprocedural medications**		
Beta blocker	1.24 (0.93 to 1.64)	0.14
Calcium channel blocker (non-dihydropyridine)	1.45 (1.10 to 1.90)	0.008
Statin	0.97 (0.72 to 1.29)	0.81
ACE-I or ARB	1.16 (0.88 to 1.52)	0.29
Antiplatelets	1.07 (0.82 to 1.40)	0.64
Warfarin or NOACs	0.90 (0.68 to 1.22)	0.52
Antiarrhythmics	0.81 (0.60 to 1.10)	0.18
**Echocardiography**		
Patent foramen ovale	0.81 (0.57 to 1.16)	0.24
*Patent foramen ovale shunt direction*	–	0.01
*No PFO	–	–
Right-to-left	1.74 (1.06–2.86)	0.03
Left-to-right	0.59 (0.36–0.94)	0.03
Bidirectional	0	0.97
Mean LAA emptying velocity	0.99 (0.98 to 0.996)	0.003
Spontaneous echo contrast (LA or LAA)	1.68 (1.24 to 2.27)	<0.001
Left atrial appendage thrombus	2.53 (1.19 to 5.37)	0.02
Severe left atrial enlargement	0.95 (0.72 to 1.27)	0.75
LV ejection fraction	1.01 (0.99 to 1.02)	0.37
Mitral regurgitation (>=moderate)	1.36 (1.02 to 1.81)	0.04
**Discharge medications**		
Beta blocker	1.30 (0.98 to 1.73)	0.07
Calcium channel blocker (non-dihydropyridine)	1.29 (0.94 to 1.78)	0.12
Statin	0.95 (0.71 to 1.26)	0.71
ACE-I or ARB	1.17 (0.90 to 1.53)	0.25
Antiplatelets	0.95 (0.72 to 1.24)	0.69
Warfarin or NOACs	1.04 (0.68 to 1.60)	0.86
Antiarrhythmics	0.89 (0.66 to 1.18)	0.41
INR at discharge	1.03 (0.87 to 1.23)	0.74

* Reference category

ACE-I, angiotensin-converting enzyme inhibitor; AF, atrial fibrillation; ARB, angiotensin-receptor blocker; CABG, coronary artery bypass grafting; CHA_2_DS_2_-VASc, congestive heart failure, hypertension, age ≥75 (doubled), diabetes mellitus, prior stroke or transient ischaemic attack (doubled), vascular disease, age 65 to 74, female; INR, international normalised ratio; LA, left atrial; LAA, left atrial appendage; LV, left ventricular; NOACs, novel oral anticoagulants; PFO, patent foramen ovale; TIA, transient ischaemic attack.

**Table 3A:** Multivariate Cox regression analysis to identify predictors of ischaemic stroke

Covariates	Ischaemic stroke 214 (7.0%)	
			HR (95% CI)	P value
*Patent foramen ovale*	0.82 (0.57 to 1.18)	0.50
CHA_2_DS_2_-VASc score	1.31 (1.19 to 1.43)	<0.001
Mean LAA emptying velocity ^-5^	1.04 (1.00 to 1.08)	0.07
LAA thrombus	2.07 (1.11 to 4.45)	0.03

CHA_2_DS_2_-VASc, congestive heart failure, hypertension, age ≥75 (doubled), diabetes mellitus, prior stroke or transient ischaemic attack (doubled), vascular disease, age 65 to 74, female; LAA, left atrial appendage.

### Shunt flow direction

**Figure 2:**
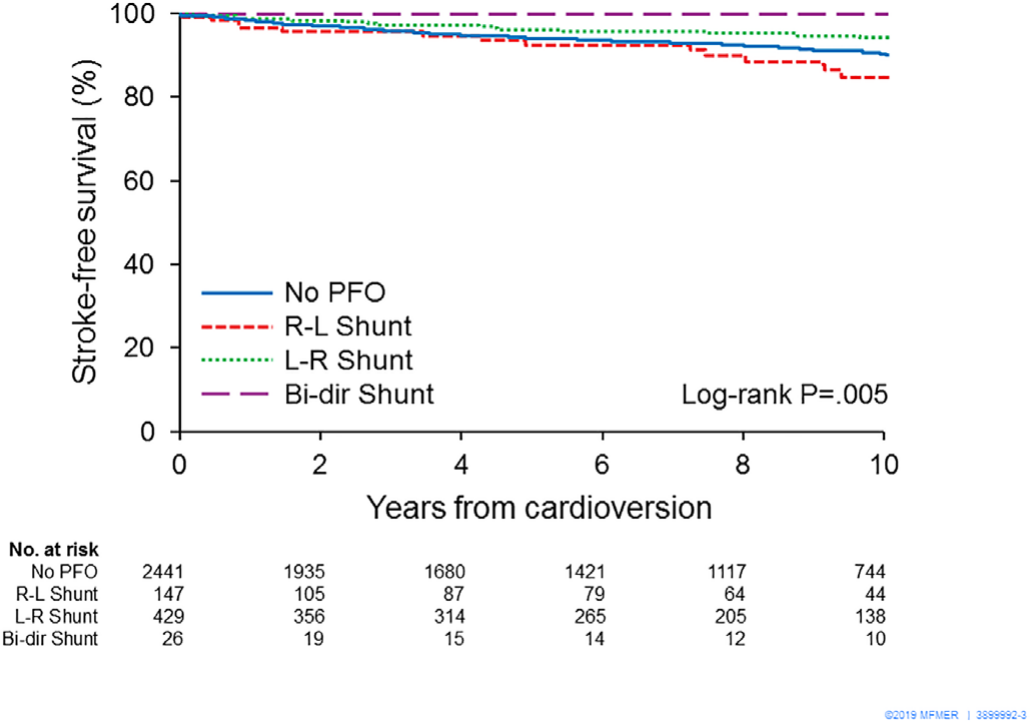
Kaplan-Meier survival analysis showing freedom from ischaemic stroke stratified by shunt flow direction versus no PFO. Patients with right-to-left shunting had a lower probability of stroke-free survival and those with left-to-right shunting had a higher stroke-free survival than did patients without PFO (log-rank p=0.005). Bi-dir, bidirectional; L-R, left-to-right; PFO, patent foramen ovale; R-L, right-to-left.

Since the PFO shunt flow direction was observed to be left-to-right in the majority of this AF cohort, we also performed multivariate Cox regression analysis to examine the impact of PFO directionality on the risk of stroke. After adjusting for variables that were significant in univariate analysis, PFO shunt direction had an independent impact on the risk of stroke: (right-to-left, HR: 1.91 (1.16 to 3.16); left-to-right, HR: 0.58 (0.36 to 0.93) (table 3B). Kaplan-Meier survival analysis showed a lower probability of stroke-free survival in patients with right-to-left shunting and a higher stroke-free survival in those with left-to-right shunting compared with patients without PFO (log-rank p=0.005). The group with bidirectional shunt was relatively small (n=26) and had a limited number of events during follow-up, making it difficult to generate any clinically reliable conclusion (figure 2).

**Table 3B:** Multivariate Cox regression analysis to assess the independent impact of PFO shunt flow direction on the risk of ischaemic stroke

Covariates	Ischaemic stroke 214 (7.0%)	
	HR (95% CI)	P value
**Patent foramen ovale shunt direction**		
Right-to-left	1.91 (1.16 to 3.16)	0.01
Left-to-right	0.58 (0.36 to 0.93)	0.02
Bidirectional	0	0.97
No PFO*	–	**–**
CHA_2_DS_2_-VASc score	1.31 (1.20 to 1.43)	<0.001
Mean LAA emptying velocity ^-5^	1.03 (0.99 to 1.07)	0.08
LAA thrombus	1.75 (1.09 to 3.19)	0.03

* denotes reference category

CHA_2_DS_2_-VASc, congestive heart failure, hypertension, age ≥75 (doubled), diabetes mellitus, prior stroke or transient ischaemic attack (doubled), vascular disease, age 65 to 74, female; LAA, left atrial appendage; PFO, patent foramen ovale.

## Discussion

### Main findings

To our knowledge, this is the first study to date to assess the incremental effect of PFO on the risk of adverse cerebrovascular events in patients with AF. In this study of patients with AF referred for TEE-guided electrical cardioversion, the principal findings are (1) that the presence of a concurrent PFO does not increase the risk of ischaemic stroke in patients with AF, (2) only traditional risk factors such as CHA_2_DS_2_-VASc score, LAA thrombus, in addition to shunt flow direction were independently associated with ischaemic stroke, (3) unprovoked left-to-right shunting at the atrial level predominated at baseline and had a protective effect against stroke in patients with AF while right-to-left shunting was associated with an increased risk of stroke in patients with AF.

The prevalence of PFO in the general population has been estimated to be 25% to 33%.^3 13 14^ In our study, the prevalence of PFO was 20%, which is slightly lower than that in the general population, but comparable with that of previous studies showing that the frequency of PFO declines steadily with advancing age,^14 15^ likely due to higher prevalence of cardiovascular conditions that can increase LA pressure in the AF population.^10^ Our results also validate previous findings on risk factors for ischaemic stroke and suggest that asymptomatic PFOs, specifically with left-to-right shunting, in anticoagulated patients with AF are of uncertain clinical significance and do not appear to require immediate intervention beyond standard therapy for AF.

### Comparison with previous studies

The scientific literature is discordant on the relationship between PFO and ischaemic stroke. Although prior studies have shown an association of PFO with stroke in young patients without identifiable cause,^3 14^ the impact of PFO on stroke risk in the elderly population remains controversial. Some earlier studies have shown a significant increase in the risk of stroke,^15 16^ whereas others have not.^2 17^ Di Tullio *et al*^17^ examined the relationship between PFO and risk of ischaemic stroke in the Northern Manhattan Study cohort using transthoracic echocardiography with contrast injection in 1100 stroke-free subjects over 39 years (mean 68.7±10 years). Participants were followed up annually to ascertain the outcome of ischaemic stroke. The observed prevalence of PFO was 14.9%. Similar to our study (mean age 69.4±12.2 years), during a mean follow-up of 79.7±28.0 months, the stroke-free survival did not differ between patients with and without a PFO. Likewise, Meissner *et al*^2^ showed that PFO was not an independent risk factor for future cerebrovascular events in the SPARC study, a prospective, population-based study of 577 randomly sampled participants at least 45 years old (mean, 70±13 years).

By contrast, Homma *et al*^16^ retrospectively assessed the effect of age on the risk of recurrent ischaemic stroke or death over a 2-year follow-up period in a subset of 250 patients enrolled in the PFO in Cryptogenic Stroke Study who were treated medically for cryptogenic stroke. They showed a significant correlation between the presence of PFO and the risk of recurrent stroke in patients 65 years and older, but not in patients less than <55 or in patients 55 to 64 years. However, this study was limited by small sample size and low number of clinical events.

### Proposed mechanisms

#### Role of PFO shunt flow direction

It must be emphasised that PFO-related shunt directionality may play a major role in the risk of stroke. Prior studies have shown that patients with cryptogenic stroke have larger PFO and more extensive right-to-left shunt flow than patients with stroke of determined cause.^8 18^Although our study confirmed the impact of PFO-related shunt directionality, the dominant direction of interatrial shunting at baseline (ie, unprovoked) observed in this AF population was left-to-right, presumably due to the left-to-right atrial pressure gradient through the PFO, consistent with the higher mean LAAEV observed in the group with PFO versus those without PFO. Indeed, patients with AF are known to have an increased burden of cardiovascular diseases, such as hypertension, and peripheral vascular diseases and are therefore more vulnerable to developing diastolic dysfunction and elevated LA pressure.^10 11 19^

#### Lack of association of PFO with ischaemic stroke

After birth, the foramen ovale closes functionally as pulmonary vascular resistance decreases and allows pressure in the LA to exceed that in the right atrium. A similar process appears to manifest in the elderly due to changes in loading conditions.^1^ It is also possible that elevated LA pressure due to loss of LV compliance, which often coexists with AF in the elderly population,^20^ could theoretically limit right-to-left shunting. Subsequent late spontaneous fusion or functional closure of PFOs in this patient population could also paradoxically eliminate the risk of paradoxical embolism as a mechanism of stroke.^21^

#### Role of anticoagulation

Previous studies have shown that treatment of PFO with OAC therapy may mitigate the PFO-stroke association.^22 23^ The vast majority of patients in our study (approximately 90%) received oral anticoagulation therapy, and 50% of the patients were prescribed additional antiplatelet therapy at discharge, essentially eliminating the major mechanism for stroke (paradoxical embolisation) by preventing thrombus formation.^24^ More recent studies, including the RESPECT trial, suggest that PFO closure in patients with no competitive stroke risks prevents strokes equally well but not better than OAC,^25^ but it avoids the bleeding risk of OAC and may be the treatment of choice.^26^ PFO closure may be another viable therapeutic option as it can confer collateral benefits, particularly in such patients with right-to-left shunt as well as those with OSA, including improved sleep-disordered breathing and nocturnal arterial oxygenation, reduced nocturnal blood pressure, attenuation of endothelial dysfunction and vascular stiffening and improvement of left ventricular diastolic function.^27 28^

### Clinical implications

In the current study, the finding of no association of PFO with stroke in patients with AF suggests that adequate OAC with or without concurrent antiplatelet therapy may suffice to mitigate the purported PFO-stroke association. Although OAC is a physiologically attractive medical therapy to prevent recurrent stroke in patients with a PFO, it is not clear whether PFOs detected incidentally in patients without prior history of embolic events comprise an indication for preventive treatment. The more interesting question of whether a PFO may increase the stroke risk in AF patients who are not on anticoagulation could not be addressed by this study.

## Limitations

Our data are based on a single-centre experience, and a number of limitations should be taken into consideration for a correct interpretation of results. The study was non-randomised and therefore we cannot preclude the presence of unidentified confounders. However, the study data were collected prospectively from both groups, and all the TEE-guided electrical cardioversions were performed in the same time period, therefore, confounding factors such as advances in technology and operator experience were eliminated. Although both groups were similarly studied by TEE with the use of colour Doppler and saline contrast imaging, clinical risk factors that could potentially enhance the effect of PFO on the risk of paradoxical embolism, such as PFO size,^8 18 24^ atrial septal aneurysm,^29^ deep vein thrombosis^30^ and hypercoagulability^24^ were not recorded. However, this limitation would likely bias in favour of an association of PFO with stroke. It is also possible that PFO was underdiagnosed in the non-PFO group, therefore biassing the study toward the null hypothesis. However, the two groups of patients analysed were homogeneous and similar imaging methodology and diagnostic criteria were employed across all patients, such that the effect should be balanced between both groups. Despite the very high rate of OAC prescription at discharge, the rate of stroke could be underestimated as information on anticoagulation status during follow-up was not collected. However, both groups had similar CHA_2_DS_2_-VASC score and were therefore equally likely to continue OAC therapy during follow-up.

## Conclusions

The presence of PFO in patients with AF was not associated with embolic risk beyond those with right-to-left shunt and other established risk factors in this predominantly anticoagulated AF population. Large-scale studies are needed to further examine the impact of PFO and shunt flow direction on clinical outcomes in AF patients.
